# Bioleaching of iron from laterite soil using an isolated *Acidithiobacillus ferrooxidans* strain and application of leached laterite iron as Fenton’s catalyst in selective herbicide degradation

**DOI:** 10.1371/journal.pone.0243444

**Published:** 2021-03-30

**Authors:** Bhaskar S, Basavaraju Manu, Sreenivasa M Y

**Affiliations:** 1 Department of Civil Engineering, National Institute of Technology Karnataka, Surathkal, Mangalore, India; 2 Department of Civil Engineering, National Institute of Technology Karnataka, Surathkal, Mangalore, India; 3 Department of Studies in Microbiology, University of Mysore, Mysuru, Karnataka, India; Indian Institute of Technology Patna, INDIA

## Abstract

A novel isolated strain *Acidithiobacillus ferrooxidans* BMSNITK17 has been investigated for its bioleaching potential from lateritic soil and the results are presented. System conditions like pH, feed mineral particle size, pulp density, temperature, rotor speed influences bioleaching potential of *Acidithiobcillus ferrooxidans* BMSNITK17 in leaching out iron from laterite soil. Effect of sulfate addition on bioleaching efficiency is studied. The bioleached laterite iron (BLFe’s) on evaluation for its catalytic role in Fenton’s oxidation for the degradation of ametryn and dicamba exhibits 94.24% of ametryn degradation and 92.45% of dicamba degradation efficiency. Fenton’s oxidation performed well with the acidic pH 3. The study confirms the role of *Acidithiobacillus ferrooxidans* in leaching iron from lateritic ore and the usage of bioleached lateritic iron as catalyst in the Fenton’s Oxidation.

## 1. Introduction

Worldwide application of herbicide to increase the significant yield of agricultural production by controlling weeds and pests may in turn have an adverse effect with environmental pollution. It is the bioaccumulation, lipophilic property, long half-life and large range transport that make herbicide more persistent in the environment. Persistent Organic Pesticides (POP’s) are the class of herbicides which includes organochlorides that pose hazard to the environment. Pesticides like aldrin, isobenzane, pentachloro phenol are likely to be classified as highly hazardous exposure to which pose neurotoxicity and DNA damage. Most of the herbicides are likely to present for a longer period deteriorating environmental quality [1–3]. Ametryn, an herbicide belongs to triazine class have an adverse effect on environment in spite of its vast application for sugarcane, corn and pineapple crops. Dicamba is another class of benzoic acid herbicide formulated to substitute atrazine compounds for the control of woody plants and broad leaf weeds [2, 4–7] Movement and persistence of this herbicide in the environment depends on many factors which make it difficult to predict [6].

Advanced oxidation process is one of the promising technologies in the degradation of hazardous herbicides among which Fenton’s oxidation proves to be much effective [8, 9]. Use of H_2_O_2_ for the degradation of triazine compounds has been proved efficient and many researches in this regard have shown that Fenton’s oxidation process is effective in the degradation of organic pollutants [10–12]. Iron playing catalytic role in the production of hydroxyl radicals attacks the target compound and increases the efficiency of process reducing the treatment time. Larson and co-workers (1991) investigated the catalytic action of ferric in the photooxidation of triazine compounds claiming the use of ferric enhances the degradation by two to three orders of magnitude. Use of iron as a catalyst either in ferric or ferrous form as a catalyst in Fenton’s oxidation adds up to the treatment in large quantity. Industrial application of Fenton’s treatment at large scale fails with the investment to commercial iron and hydrogen peroxide. To overcome this research has been done to replace commercial iron with laterite iron extracted using chemical methods [13–17].

Chemical extraction of iron from laterite soil being expensive fails at large scale application. Bioengineering in this regard has made its trustworthy contribution towards mineral processing. *Acidithiobacillus ferrooxidans*, chemolithotrophic acidophilic gram negative bacteria which relay on iron oxidation for its energy source and can reduce sulphur is extensively used as a key organism in the bioleaching process [18]. The process is widely adapted for the recovery of gold, copper zinc etc. at large scale [19–23]. Chang and team successfully attempted to leach out iron from laterite nickel ore by acid leach liquor method [24]. Recent advances in bioleaching include recovery of zinc and other valuable metals using an electro dialysis system and indirect method of leaching by means of metabolites from the various bacteria [25, 26]. Present investigation focusses on the extraction of iron from lateritic soil using an isolated bacterial strain *Acidithiobacillus ferrooxidans* BMSNITK17 and its potential application as a substitution for commercial iron in the Fenton’s treatment for the degradation of ametryn and dicamba which has not been studied yet.

## 2. Material and methods

### 2.1 Bioleaching of iron from laterite soil

Laterite soil samples were collected from the NITK campus, Surathkal, Karnataka, India. Soil samples were processed for required size and sterilized by steam autoclaving. Modified 9K media was used for bioleaching studies without ferrous iron supplement to which laterite soil was added to makeup definite volume of mixture [27]. 10 ml of an isolated *Acidithiobacillus ferrooxidans* BMSNITK17 (Accession No. MG27180) inoculums with a cell count 1.0 x 10^7^ cells/ml was then added to this mixture to initiate the leaching studies [28]. Experiments were conducted with different operational conditions to study the effect of influencing parameters like Pulp density (2.5%, 5%, and 10%), Temperature (25°C, 30°C, 35°C, and 40°C), Shake flask speed (100 RPM, 180 RPM, and 250 RPM), pH (1.5, 2.0, 2.5, and 3.0) and Particle size (2.36 mm, 300micron, 150micron, 75micron, 53micron). All the experiments were conducted in dual with sterile conical flasks on incubator shaker for eight days.

### 2.2 Bioleached laterite iron catalyzed Fenton’s oxidation of selective herbicides

Iron catalyzed Fenton’s oxidation of ametryn and dicamba were carried out with 5 mg/L and 100 mg/L of initial herbicide concentration to mimic the field concentration [29]. Ametryn and dicamba solution prepared in the laboratory by adding 5 mg of ametryn to 1000 ml of distilled water and 100 mg of dicamba to 1000 ml of distilled water respectively. Both the solution was separately taken in 5L conical flask and kept in a magnetic stirrer until added chemicals gets dissolved completely. 100 ml of prepared ametryn and dicamba solution were taken in a conical flask separately to which bioleached laterite iron solution was added in an incremental rate. The solution was adjusted to pH 3 using 1N H_2_SO_4_ and allowed 10 minutes prior to H_2_O_2_ addition to ensure that the addition of H_2_O_2_ triggers the initiation of reaction. Investigation was conducted with experimental conditions of bioleached laterite iron (10 mg/L, 20 mg/L, 30 mg/L and 40 mg/L for dicamba and 3 mg/L, 4 mg/L, 5 mg/L and 6 mg/L for ametryn) and H_2_O_2_ dosage (100 mg/L, 200 mg/L, 300 mg/L and 400 mg/L for dicamba and 30 mg/L, 40 mg/L, 50 mg/L and 60 mg/L for ametryn). Samples were drawn at regular intervals for analysis. During sampling, each time 1ml of sodium thiosulphate was added to arrest the reaction [15]. All the experimental analysis was conducted in triplicates.

### 2.3 Scanning Electron Microscopic (SEM) analysis

Morphological feature of laterite soil before and after bioleaching was studied with SEM. Change in structural and mineralogical composition during the leaching was studied using a S-3400N scanning electron microscope. Samples were dehydrated and dried before being mounted on specimen. After being gold coated and examined under a scanning electron microscope (Hitachi S-3400, Japan) at 15 kV at 500 SE.

### 2.4 Analytical procedure

Ametryn and Dicamba concentration was measured by a high-performance liquid chromatograph (HPLC) Agilent 1200 make [28]. Chemical oxygen demand (COD) was measured by colorimetric method as per 5220D of Standard Methods for Examination of Water and Wastewater [30]. The H_2_O_2_ consumption was measured using UV-Vis spectrophotometer [31]. Concentration of ferric iron was measured by potassium thiocynate method using UV-Spectophotometer [32]. Concentration of ferrous iron was measured by 1,10 phenonthroline method [30]. The pH was monitored by digital pH meter (HANNA make). Chloride content was monitored by argentometric method. Oxidation and reduction potential were measured with Redox meter (EUTECH make).

## 3. Results and discussion

### 3.1 Effect of shake flask speed

**Fig 1:**
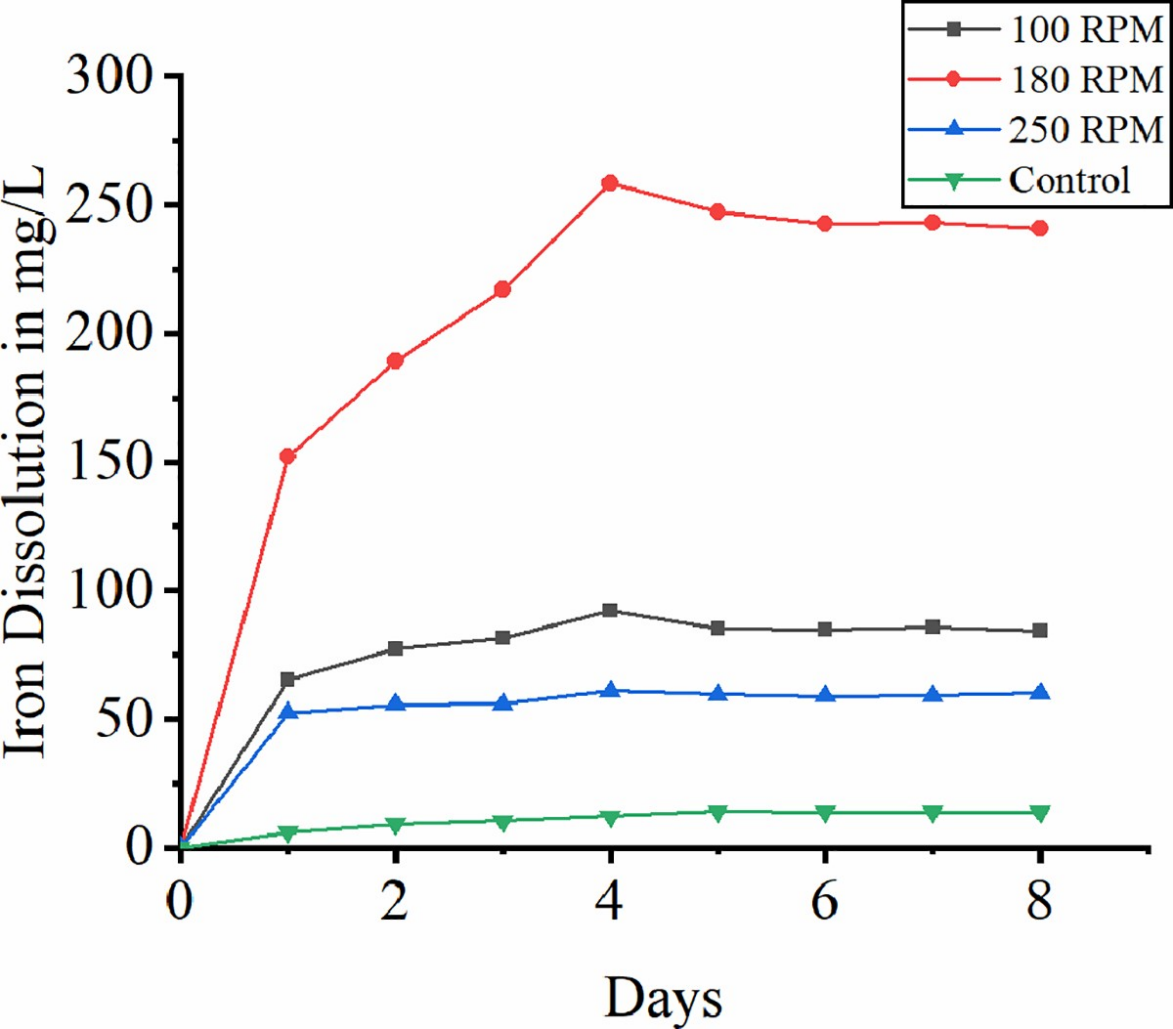
Effect of shake flask speed on bioleaching of iron.

Agitation speed in the bioreactor is of much concern with process engineering which corresponds to shake flask speed at lab scale shake flask bioleaching studies. In the present study the effect of shake flask speed at different variants like 100rpm, 180rpm and 250rpm was studied. Fig 1 shows the rate of iron dissolution at different shake flask speed. At 180rpm the rate of dissolution is comparatively high since the agitation at this speed holds the bacterial suspension in contact with ore allowing ore particles not to settle down at the bottom. In unbaffled shake flask it is the flow pattern which affects the rate of leaching [33]. Buchs (2001) study confirms that the laminar flow at 100 rpm and 180 rpm keeps the suspension in contact with bacteria while at 300 rpm the rate of dissolution is low because of turbulence that occurs at this speed [33]. Higher shear force at high speed of shake flask might be one of the causes for cell detachment to ore surface and cell rupturing [34]. In the present study the initial pH-maintained decreases initially indicating the proton consumption due to better oxidation rate. This was supported by higher redox potential value in first 4 days of the study. Maximum iron dissolution of 258.3 mg/L was observed at 180 rpm on fourth day of leaching studies. The precipitation of ferric iron due to hydrolysis and the maximum utilization of ferrous iron leached out from the laterite by the bacteria might be the reason for the decrease in iron dissolution after fourth day.

### 3.2 Effect of pH

**Fig 2:**
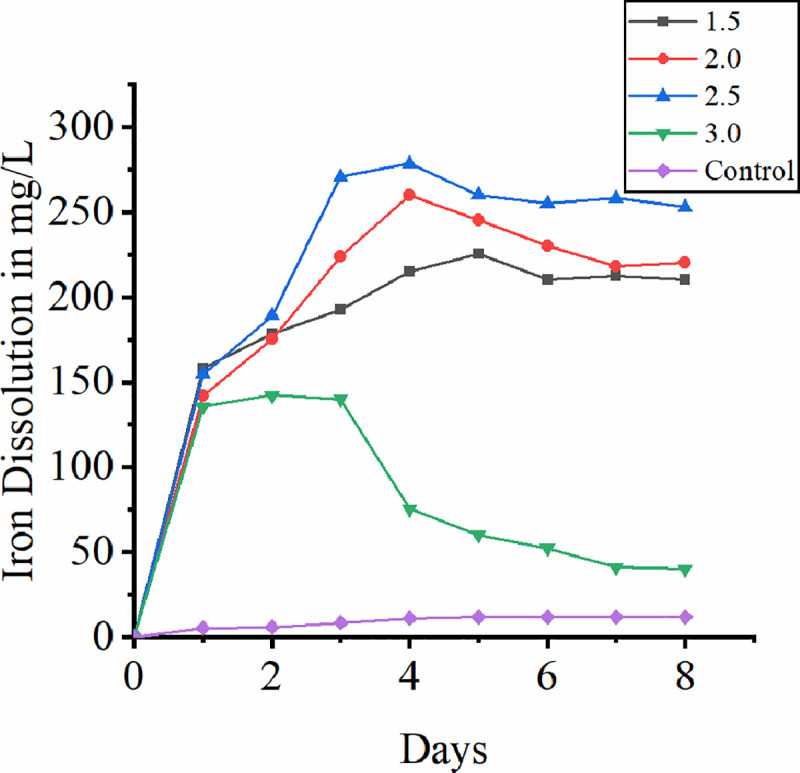
Effect of pH on bioleaching of iron.

pH is the prime factor for both microbial growth and biooxidation. pH value in the range of 1.5 to 3.0 gives highest biooxidation rate [35]. In the present study higher redox potential in the range of 550–600 mv supports this statement. Flower and coworkers (1999) claim that even on reduction of pH from 1.7 to 1.3 the rate of iron dissolution increases. This might be due to chemical dissolution of iron into solution at lower pH [36]. Fig 2 dissipates the iron dissolution from lateritic soil at different pH. In the present study maximum iron dissolution of 281.0 mg/L was observed at pH 2.5. Thus, the result obtained is within the optimum range of pH for bacterial metal dissolution process. In the first four days of leaching drop in initial pH was observed. This might be due to ferrous oxidation resulting in more proton consumption [18, 19, 37]. Caranza and Paleneia (1996) claim that pH lower than 0.8 inhibits bacterial growth in spite of acidophilic mode of habitation in leaching studies which was supported by Marrero and team on their experimentation with *Acidithiobacillus ferrooxidans* later indicating the sensitivity of species to low pH [38, 39]. Battaglia and his coworkers (1994) stated that bacterial growth is usually inhibited at pH 1.5. In contrast to this, in the present study we observed a significant iron dissolution rate at pH 1.5. The reason for this is attributed to this is leaching occurs by dissolution at lower pH which accounts for iron leaching that is release of ferric iron from solid phase even in the absence of bacterial action [39]. To check this uninnoculated control was kept at pH 1.5 in which dissolution of iron was observed confirming the iron leaching in small quantity. Initial drop in pH during the study indicates the biooxidation of iron while the later increase indicates ferric iron precipitates by hydrolysis and also shifts in iron oxidation to sulfur reduction [40]. Drop in pH may also be due to bacterial growth whereas increase in pH indicates no further cell growth. In the present study, during the first four days the concentration of iron dissolution is high indicating the release of ferric iron by means of proton attack. This observation is in consistent with the previous study [39].

### 3.3 Effect of pulp density

**Fig 3:**
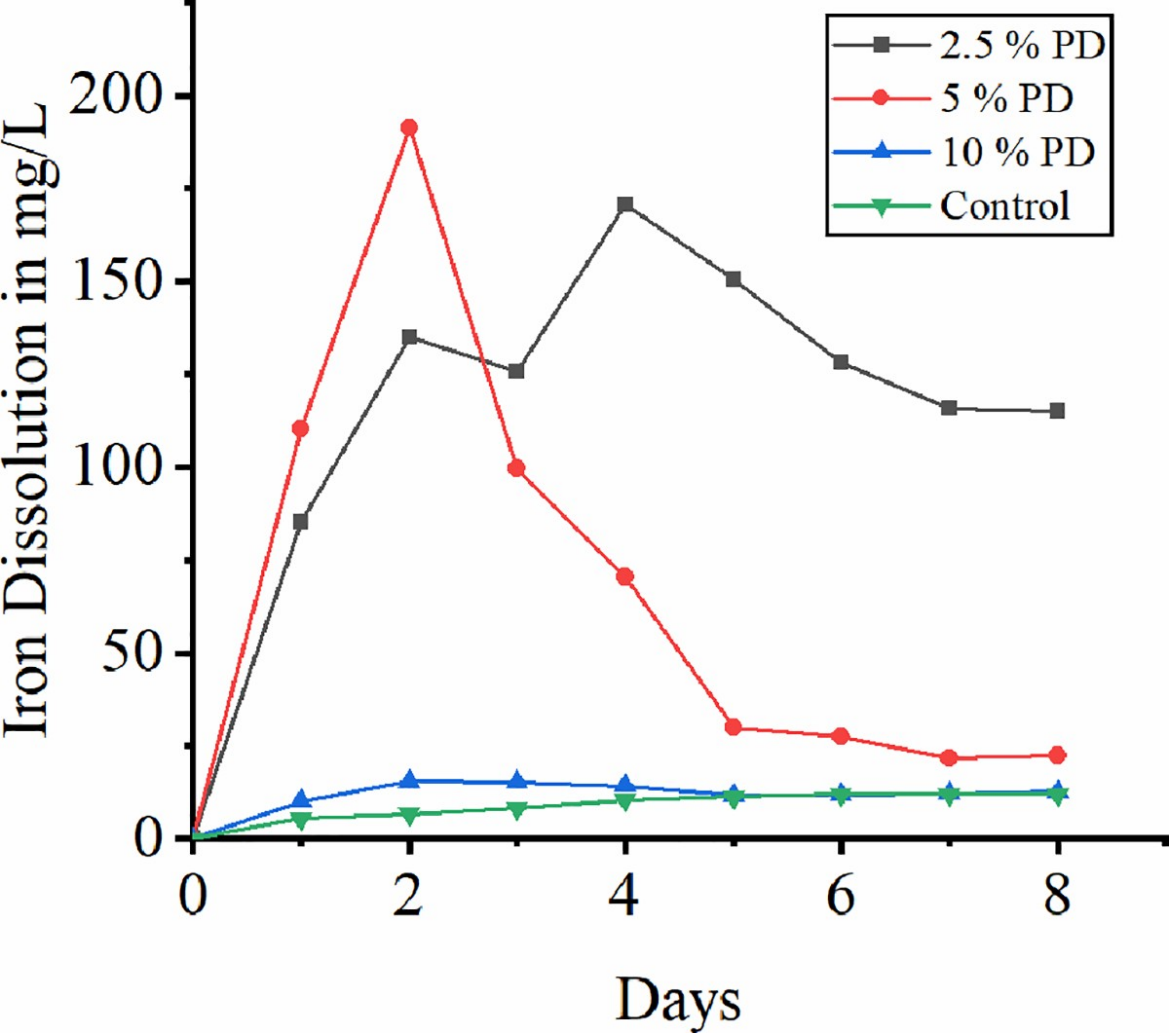
Effect of pulp density on bioleaching of iron.

Fig 3 represents the iron dissolution rate at different pulp densities. Mousavi and team (2005) found the reduction in copper bioleaching on increase in pulp density from 10% to 20% and claims at high pulp density the oxygen supply is limited due to gas liquid mass transfer rendering death of microbial cells. In the present study at pulp density 5% maximum iron dissolution of 191.36 mg/L was observed. However, on increase in the pulp density to 10% significant iron dissolution has not been found. Since lateritic soil contains more iron in the form of ferric than ferrous it was the ferric iron load on bacteria hinders the rate of iron dissolution at high pulp density. Pulp density can be kept higher with respect to low ferric iron concentration [38]. This investigation deals with the leaching of iron from the laterite soil which has more ferric iron content than ferrous. Hence maintaining ferric iron content with respect to pulp density does not comes under the scope of our study. Reduction in iron dissolution once again attributed to the oxygen and carbon dioxide limitation at high pulp density [41]. There is a sudden increase in the iron dissolution at the pulp density 5% and reaches to maximum by second day. At the pulp density of 5% complete oxidation of iron is possible and maximum leaching is observed by the third day of investigation. Iron dissolution at 10% pulp density indicates the solid load at this rate is high thereby no bacterial growth resulting in insignificant leaching [42].

### 3.4 Effect of temperature

**Fig 4:**
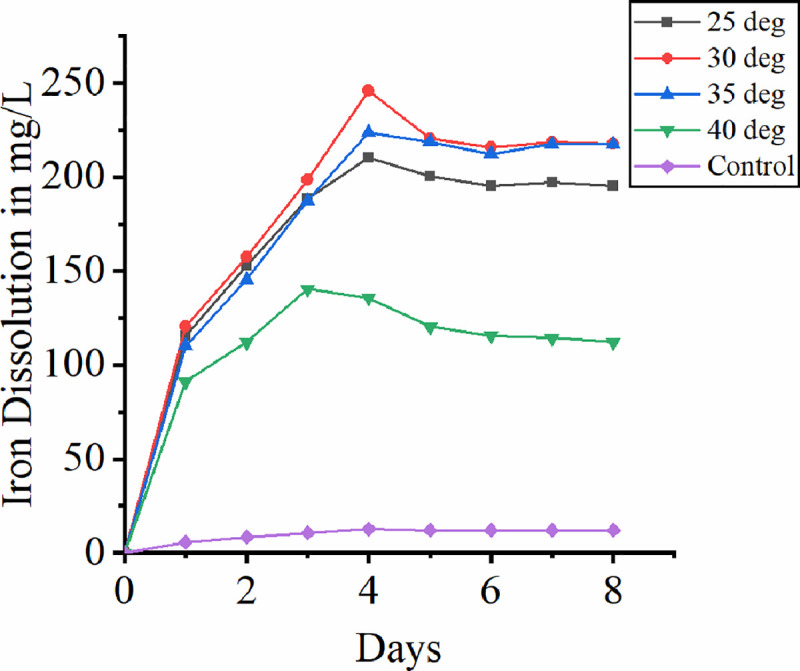
Effect of temperature on bioleaching of iron.

Many researchers have been stated that thermophiles are much more efficient in the process of bioleaching than mesophiles [43–46]. Even though the bacterial strain used here is mesophilic the broad range of temperature to which bacterial adaptation and leaching potential was evaluated in the mesophilic range (25 ^o^C– 40 ^o^C). Fig 4 shows the rate of iron dissolution at different temperature. In the present study maximum iron dissolution of 245.7 mg/L was observed at 30 ^o^C indicates the adaptation of bacterial strain to the specific temperature. At higher temperature of 40 ^o^C the rate of dissolution was reduced drastically and at lower temperature of 25 ^o^C the rate of dissolution was comparatively less. It is the smaller driving force at low temperature limiting the gas transfer rate making the leaching operation more difficult [18]. Gomez et al. (1999) observes the drop in leaching potential of mesophile on increase in temperature because of reduction in bacterial activity [47]. This was also supported by Karimi and team (2010) as they observed reduction in both iron and copper dissolution rate at 40 ^o^C [48]. At higher temperature the reduction in bioleaching potential was attributed to bacterial adaptability to temperature. From graph it can be seen the iron dissolution attains maximum with 30 ^o^C and 35 ^o^C. Since the *A*. *ferrrooxidans* strain used here is a mesophilic bacterium, the reason for maximum bioleaching at the observed temperature range is attributed to maximum bacterial activity in this range. At 40 ^o^C, the iron dissolution is observed slowly reaches stationary phase by third day and drops at a constant rate. These results are in correspondence with the leaching studies conducted by Karimi and team (2010). The reason attributed to drop in iron dissolution at 40 ^o^C is the bacterial activity slowly halts with time. In the control experiments without bacteria, the leaching rate was insignificant.

### 3.5 Effect of particle size

**Fig 5:**
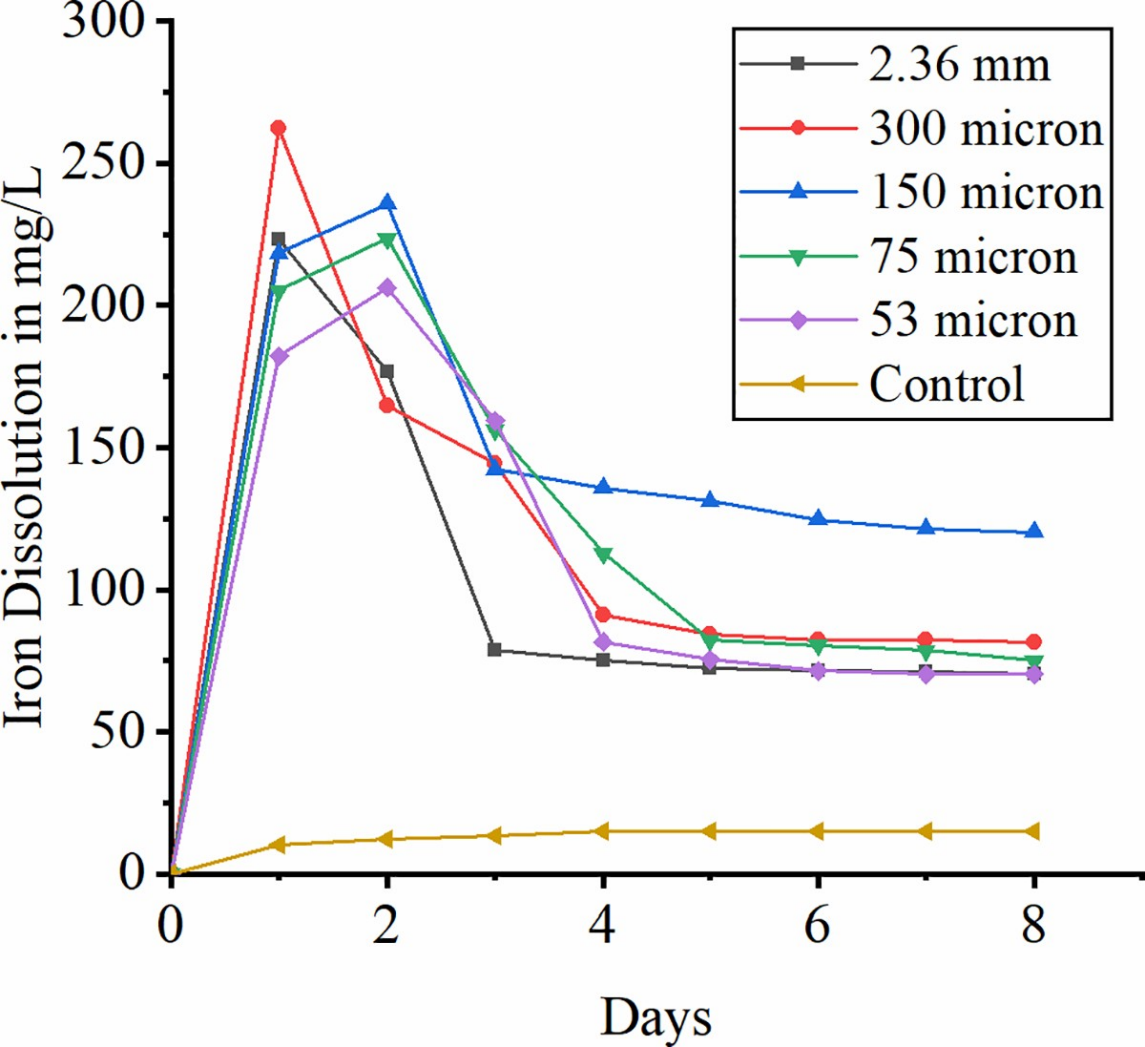
Effect of particle size on bioleaching of iron.

Particle size plays an important role in bioleaching by regulating bacterial attachment to the surface of mineral ore. Reduction in particle size increases the surface area of the particles thereby increasing the rate of metal dissolution [49–51]. Fig 5 dissipates the rate of iron dissolution in the present study. Smaller the size more the iron dissolution occurred. This may be smaller particles (less than 100μm) facilitates the uniform slurry and increased active sites of mineral [18]. Lower particle size also enhances the bacterial attachment to laterite resulting in more iron dissolution [52]. However, after first four days, the rate of dissolution is reduced because of ferric precipitation. The initial pH drops at first four days in the flasks with particle size less than 75μm and high redox potential confirms the high biooxidation rate at lesser particle size and hence more dissolution of iron. From the graph it is observed that there is a drastic increase in the iron leaching with particle size of 300 micron. Reaching to the maximum rate within 24 hours it has been observed with the sudden drop-in leaching rate. In contrast to this leaching with other particle size reaches its maximum and constantly shows the decrease at the end of the study. It is claimed that particle with size less than 75 microns favors the bacterial attachment to mineral providing more surface area. Thus, in the initial days the leaching is found to be maximum and after fourth day the leached iron on oxidizing to ferric forms precipitation which covers the mineral surface hindering the bacterial attachment thereby lowering the leaching rate.

### 3.6 Variation in ferrous and ferric iron during leaching

**Fig 6:**
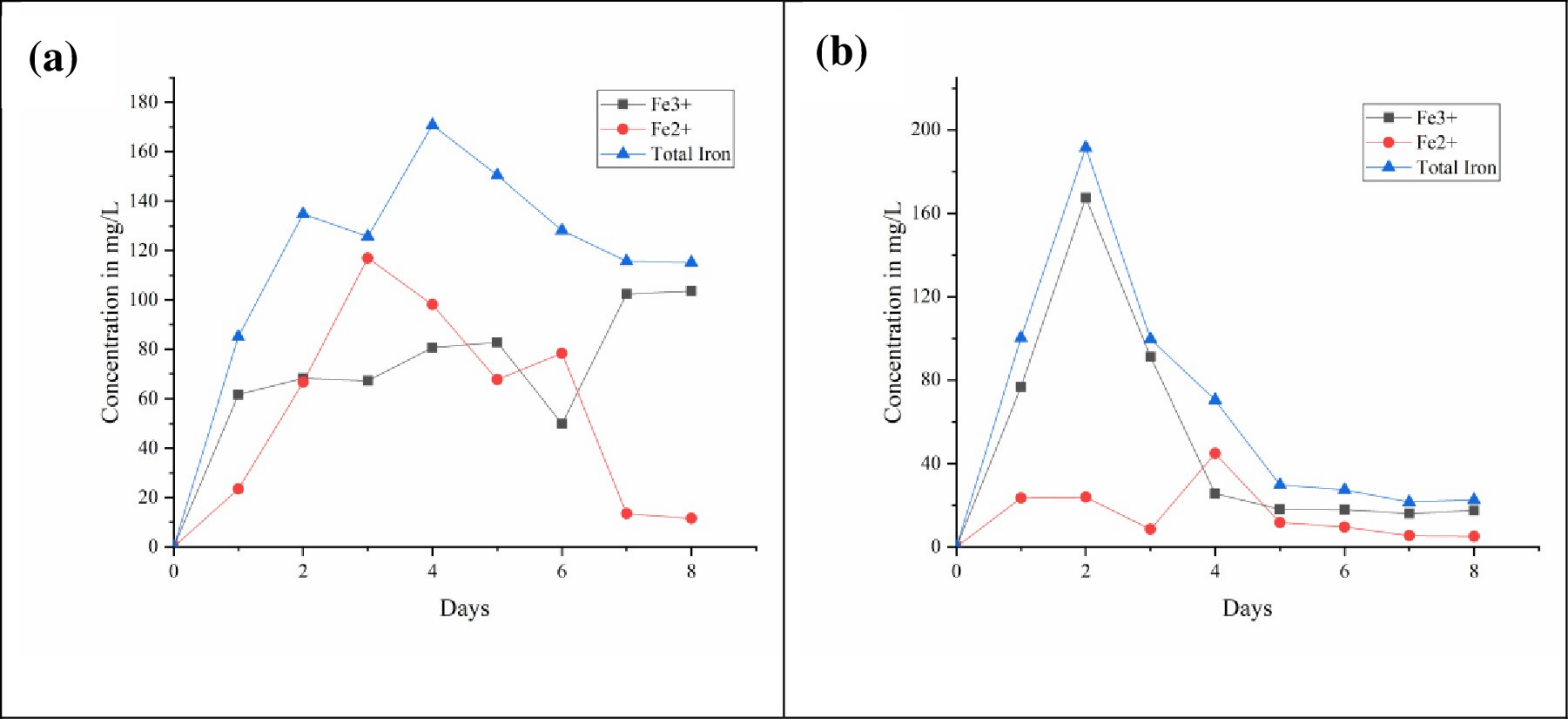
Variation of ferrous and ferric iron during bioleaching at a) 2.5% PD b) 5% PD.

Fig 6 dissipates the variation of ferrous and ferric iron during bioleaching at 2.5% and 5% pulp density. It is observed that the complete oxidation with 5% pulp density occurs by second day thereby sudden decrease in the ferric iron content in the slurry indicates the precipitation of converted iron. Daoud and Karamanev (2006), quote that on oxidation of ferrous to ferric, ferric iron on hydrolysis forms the precipitation [53]. At pulp density 2.5% the ferrous iron predominates the ferric iron indicating poor oxidation. This may be due to stationary growth of bacteria. The ferrous and ferric iron measured in the study is in consistent with redox potential and pH measured.

### 3.7 Oxidation and reduction potential and pH

**Fig 7:**
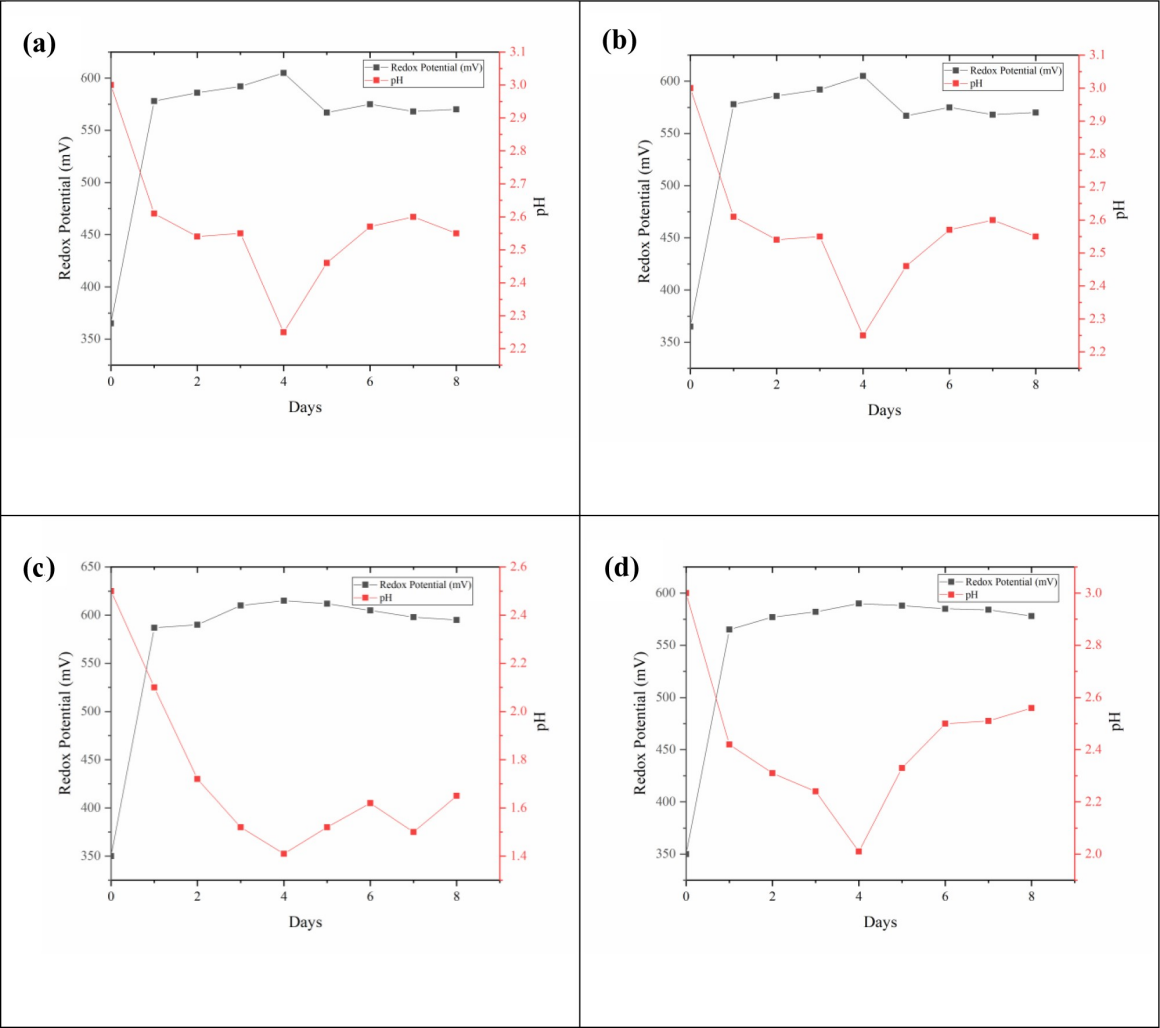
Variation of oxidation and reduction potential and pH during Bioleaching at a) 5% Pulp Density b) 180 rpm c) 2.5 pH d) 30 ^o^C.

Oxidation–Reduction potential in the study varies with pH. It has been observed that in all the conditions maintained the redox potential reaches to maximum by first day of the study. Thereafter the redox value remains constant till the end of the study indicating oxidizing environment. Fig 7 shows the variation of oxidation and reduction potential at 5% pulp density, 180 rpm shake flask speed, 2.5 pH and 30 ^o^C temperature. pH during the study decreases first indicating the bacterial activity and production of sulfuric acid. From the day four there is an increase in the pH indicating the hydrolysis predominates the bacterial action. With respect to initial pH maintained 1.5, the pH drops initially to less than 0.6 initially indicating the halt of probable bacterial action [38, 54]. Hallberg and team conducted a study on nickel lateritic leaching and claim that aerated medium results in very low metal dissolution compared to anoxic condition [55]. In contrast the declination in redox potential was observed by Marrero and team under anaerobic conditions [56]. Bacterial oxidation in the present study is narrow within the range of 2–2.5.

### 3.8 Effect of sulfate addition on bioleaching

**Fig 8:**
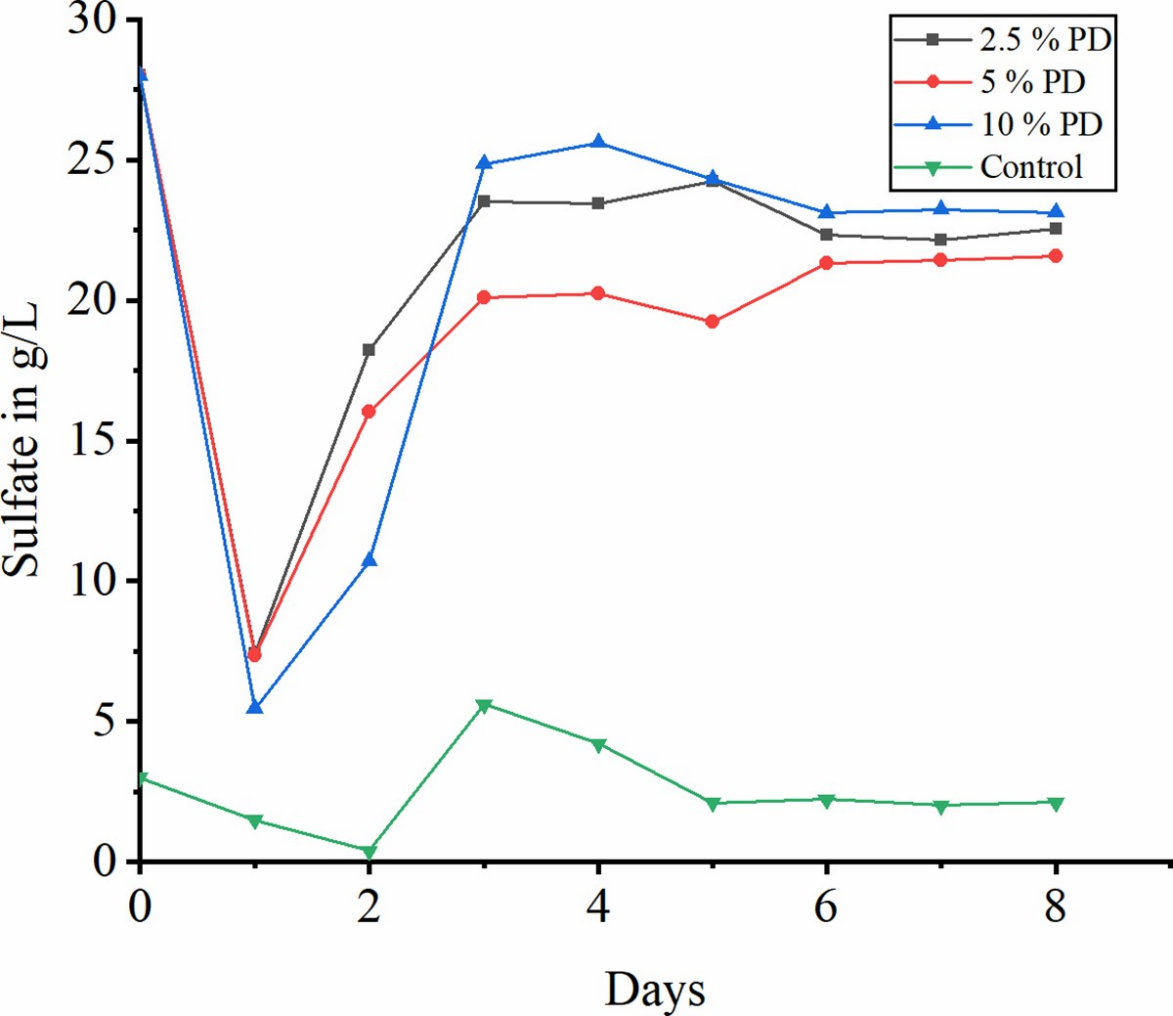
Variation of sulfate during bioleaching at different pulp densities.

There is no considerable enhancement in the bioleaching of iron on additional sulfate supply of about 28 g/L. Fig 8 dissipates the sulfate concentration during bioleaching of iron from laterite soil. It has been observed that there is a decrease in sulfate concentration provided with all pulp density by first day. In consistent to this the bacterial oxidation is found maximum both with sulfate and without sulfate supplement. Sulfate concentration in the solution increases by the fourth day gradually indicating no more sulfate consumption thereby. This might be due to inhibition of bacterial activity. The study claims that sulfate supplement does not cause the increase in the rate of leaching of iron from laterite soil. It is the gangue material present in the laterite causes the inhibition in bacterial activity. Also it is claimed that lateritic tailings shows its effective dissolution under aerobic conditions with mixed culture rather than pure culture [56, 57].

### 3.9 Iron precipitation and ferric poisoning

**Fig 9:**
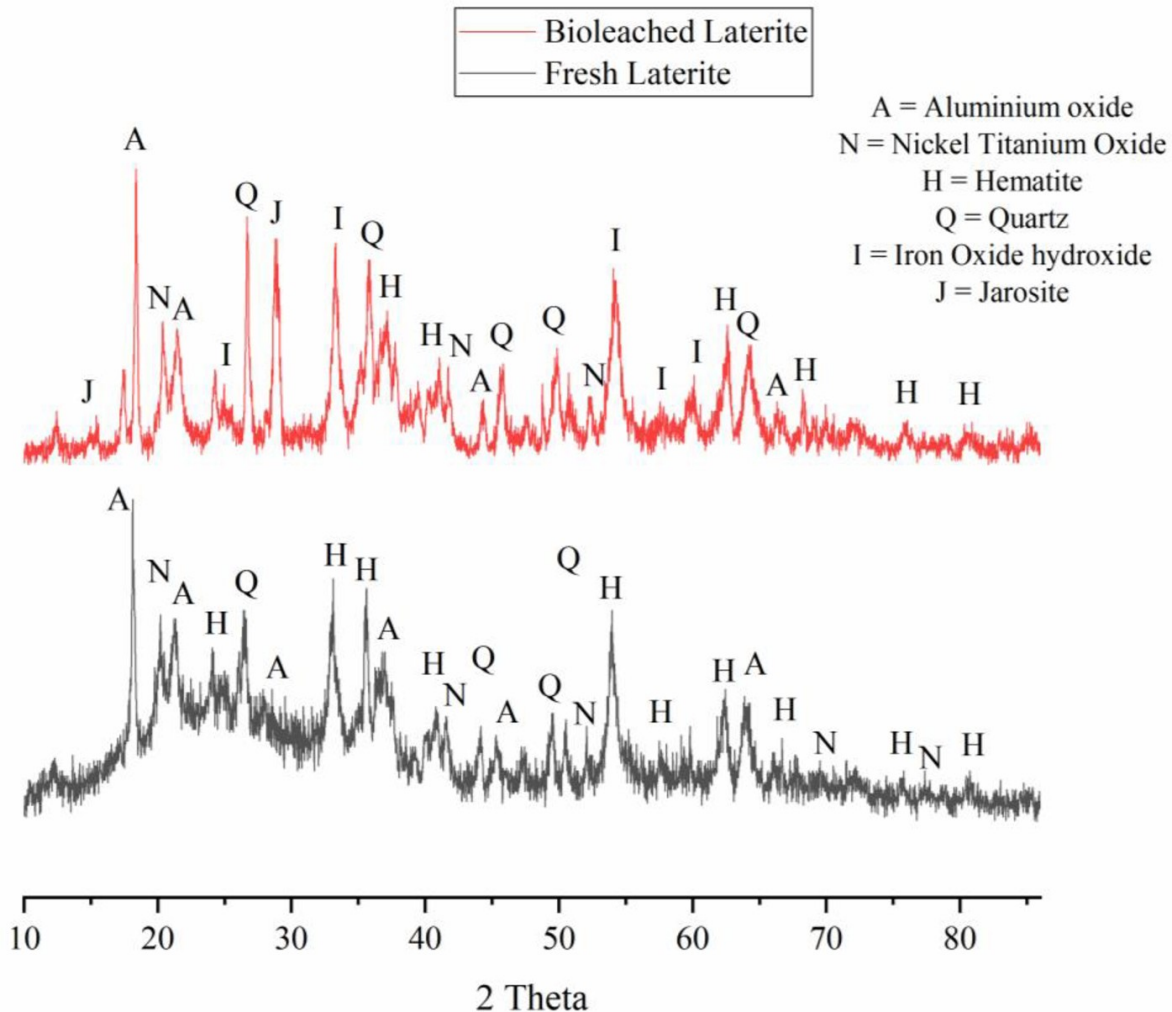
XRD pattern of study soil before and after leaching.

**Fig 10:**
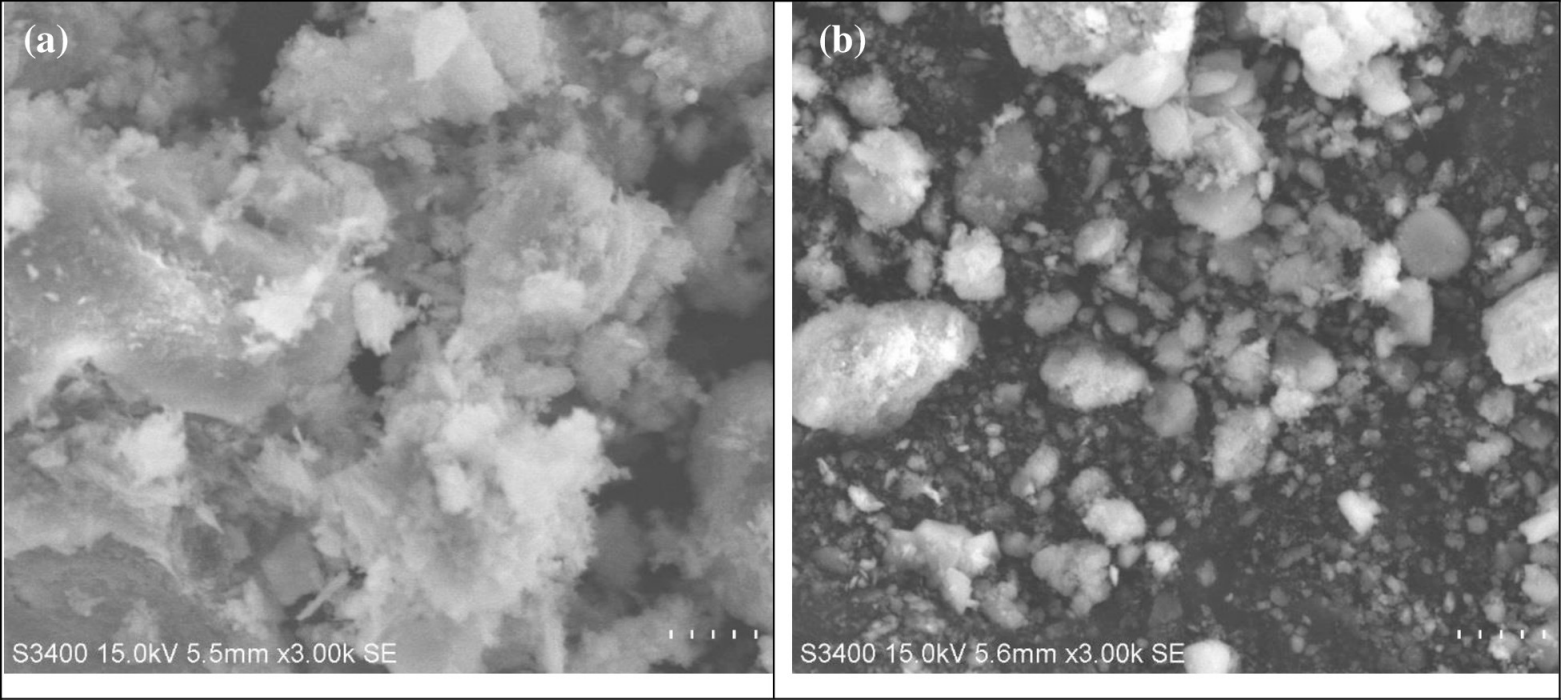
SEM images showing Laterite soil a) Before Leaching b) After Leaching.

Iron in the form of ferric possesses low solubility. On the process of biooxidation iron gets oxidized and converted into ferric iron which precipitates as jarosite. Kumar and team (1990) claims the rate of jarosite precipitation on bioleaching is proportional to the ferric concentration and inversely proportional to the hydrogen ion concentration [58]. Toro and team conducted research on precipitate formation in ferrous biooxidation and observes four phases of process in which precipitation is correlated to third phase within 48 hrs [59]. Yang and co-workers recently carried out an investigation on the formation of jarosite and claims total iron concentration in the bioleaching system increases initially due to acid dissolution and decreases thereby slowly following acid consumption and jarosite formation [60]. In the present study in pH decreases in the first two days of leaching due to proton consumption on biooxidation process and increases later indicating the jarosite precipitation due to hydrolysis. Marrero and team claims that it is it is the sensitivity of *Acidithiobacillus ferrooxidans* to high concentration of metals such as iron results in the lower dissolution rate [56, 61]. The study soil laterite contains much ferric form of iron than ferrous which again contributes to jarosite precipitation thereby hindering the mass transfer of ions into the solution [40, 62]. The layer of jarosite creates a passive barrier between the laterite and bacteria which prevents the bacterial iron access henceforth reducing its bioleaching potential [48]. Coto and co-workers studied the ferredox process targeting tropical limonitic laterites and observed that on ferrous iron oxidation process iron precipitated as jarosite and schwertmannite converts incoming ferrous iron into either a soluble ferric sulfate solution or ferric oxysulfate precipitation which will be utilized for sulfuric acid generation resulting in pH drop [63]. It is the form of iron precipitation governs the proportion of soluble and precipitated ferric iron [64]. Fig 9 shows the XRD pattern of laterite soil before and after the bioleaching. Before bioleaching the study soil exhibits the presence of Hematite (PDF No. 01-089-0596), Nickel Titanium Oxide (PDF No. 01-079-1213), Aluminum Oxide (PDF No. 00-001-0259) and quartz (PDF N-o. 01-089-8939) with broad peaks at 2Ɵ 26.81, 50.27, 55.09, 57.93, 24.32, 57.93, 64.52 whereas it is observed that there is a change in the mineral composition of the study soil with the presence of jarosite (PDF No. 00-010-0443) and Iron oxide hydroxide (PDF No. 01-076-0182) at 2Ɵ 14.89, 29.0374 and 33.29, 54.18, 62.61. Fig 10 shows SEM images of fresh and 8 days old bioleached laterite soil in which bacterial attachment has not been observed due to jarosite precipitation by 8^th^ day. Excess ferric iron concentration in the present study has negative effect on the iron dissolution rate. Kumar and team (1990) claim that the accumulation of ferric iron may poisons the bacteria. Cell lysis may occur due to ferric iron accumulation. In their study the increase in ferric concentration from 5 g/L to 20 g/L has led to the death of bacterial cell rendering ferric poisoning. In the present study ferric iron in the solution predominates ferrous form from the 1^st^ day thereby gradually leads to ferric poisoning halting the process of leaching by fourth day. Chen and co-workers conducted an assay on the effect of nickel with ferric iron on the growth of acidophilic bacteria *Acidithobacillus thiooxidans* and claims that the addition of ferric iron has a positive effect on the bacteria in inhibiting nickel toxicity [65].

### 3.10 Catalytic degradation of selective herbicides by bioleached laterite iron

**Fig 11:**
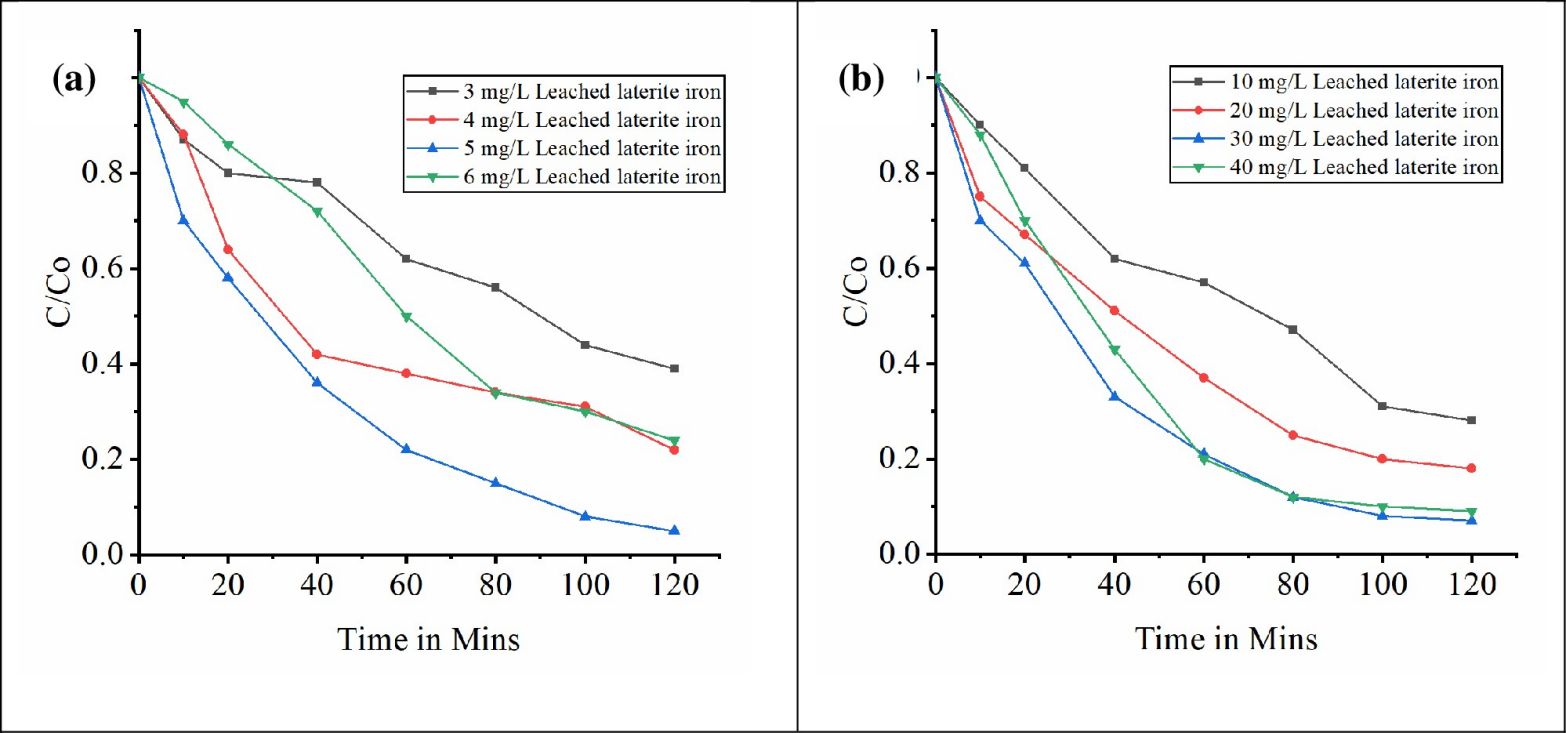
Degradation of target compound at different iron loading for a) Ametryn at 50 mg/L H_2_O_2_ b) Dicamba at 300 mg/L of H_2_O_2_.

**Fig 12:**
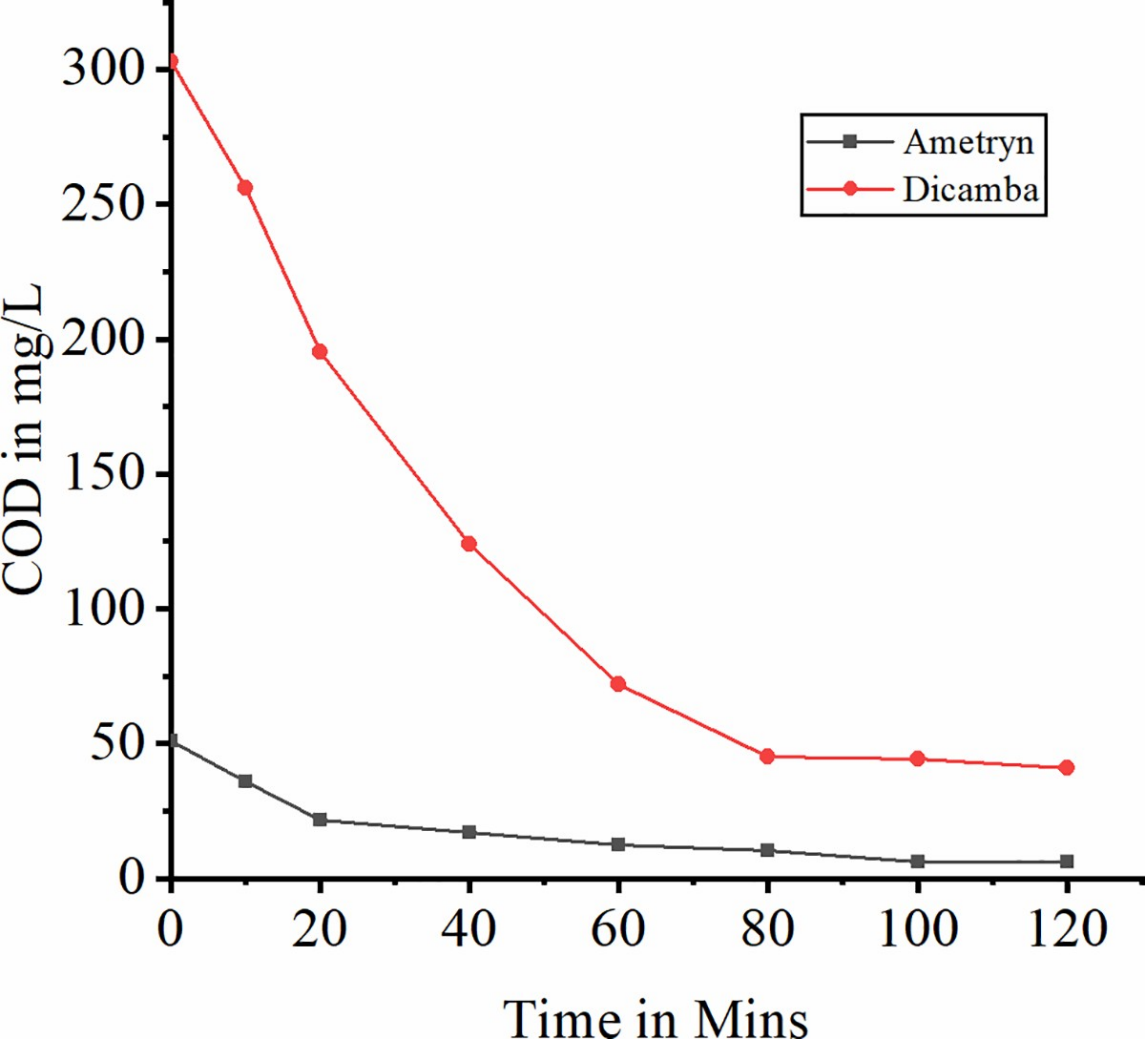
Variation of COD during the process of Fenton’s oxidation.

**Fig 13:**
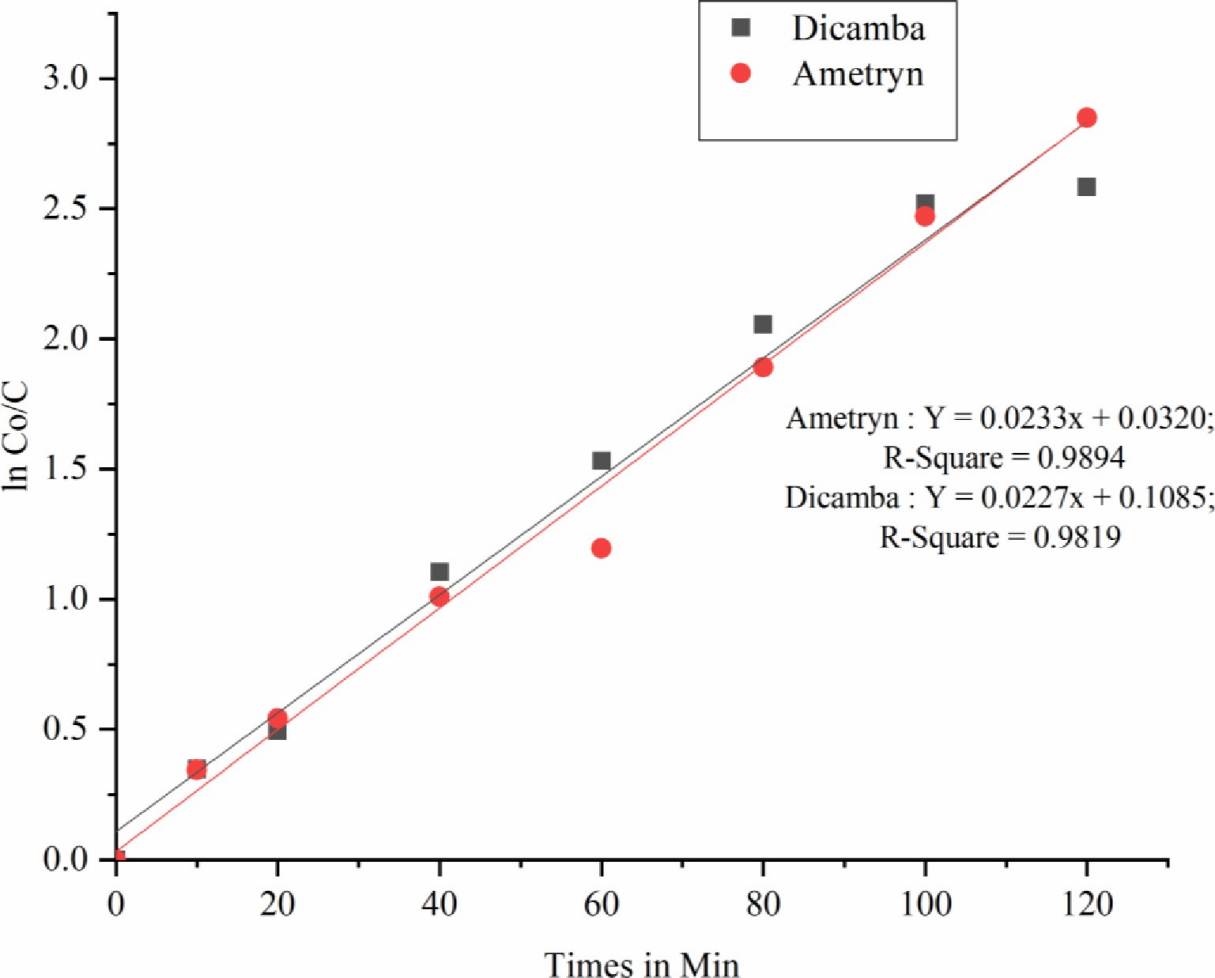
Pseudo-first order kinetic model fit ln C/C_o_ versus time.

Degradation of ametryn and dicamba by bioleached laterite iron catalyzed heterogeneous Fenton like process was investigated with initial herbicide concentration 5 mg/L and 100 mg/L respectively. The degradation however starts slowly on the addition of H_2_O_2_ initiating the reaction. High removal efficiency up to 94.24% was found with laterite iron dosage of 5 mg/L and H_2_O_2_ dosage of 50 mg/L for ametryn and 92.45% with laterite iron dosage of 30 mg/L and H_2_O_2_ dosage of 300 mg/L for dicamba at pH 3.0 and temperature 30°C. The COD removal was 88.04% and 86.4% in first 120 min respectively. About 59.02% removal for dicamba and 66.38% for ametryn was observed in the first 40 minutes indicates better oxidation for both the herbicides. Increase in laterite iron dosage more than 30 mg/L and 5 mg/L for dicamba and ametryn respectively reacts with the hydroxyl radicals hindering its role in the herbicide degradation phenomena. Similarly increase in H_2_O_2_ dosage from 100 mg/L to 200 mg/L, 300 mg/L and 400 mg/L for dicamba and 30 mg/L, 40 mg/L, 50 mg/L and 60 mg/L for ametryn did not shows any significant change with 300 mg/L and 50 mg/L bioleached laterite iron load for dicamba and ametryn respectively. This may be due to no reduction in the induction period of process on H_2_O_2_ increase [65–67]. Many research teams made a successful attempt to extract iron from laterite soil chemically by acid digestion method and studied the catalytic role of extracted laterite iron in Fenton’s process [13–15, 17]. In the present study initial pH was fixed to acidic pH because of proven process efficiency at acidic range. It was proven that at basic pH condition the ferric hydroxyl complexes may cause the drop in degradation efficiency [28, 68–70]. The effect of bioleached iron dosage on the oxidation process at maximum degradation efficiency is graphically shown in Figs 11 and 12 represents the variation of COD during the process. Among dicamba and ametryn, ametryn exhibited high removal efficiency in first 120 mins indicating its susceptibility for the Fenton’s process. The presence of methylthio group in the ametryn makes it more amenable for hydroxyl ion attack [28]. Both the degradation of dicamba and ametryn follows pseudo first order reaction. Fig 13 shows the kinetic fit. Overview of Fenton’s degradation of ametryn and dicamba is tabulated in Table 1.

**Table 1:** Fenton’s oxidation of selective herbicides.

Target Compound	First order kinetic regression R^2^	First order rate constant k (min^-1^)	Degradation efficiency (%)	COD Removal rate (%)
Ametryn	y = 0.0233 x (0.989)	0.032	94.24	88.04
Dicamba	y = 0.0227 x (0.981)	0.108	92.45	86.40

## 4. Conclusions

A novel isolated iron oxidizing bacterial strain *Acidithiobacillus ferrooxidans* BMSNITK17 role in iron leaching from laterite soil was investigated and found that the process of leaching lasts for few days. It is observed that the strain alone has not shown satisfactory leaching which is also supported by previous studies. Shake flask speed of 180 rpm was found to be optimum since at this rpm holds the bacteria in a solution in suspension providing a better contact of bacteria with the mineral ore. pH in the range of 2.5–3.0 and temperature of 25 ^o^C—30 ^o^C were found to be optimum for bioleaching because of bacterial adaptation at this pH and temperature. Pulp density of 5% was found to be optimum in iron dissolution as this provides the better gas transfer allowing the microbial metabolism to occur and particle size in the range 150–75 μm found to be optimum as higher particle size does not provides the bacterial attachment to mineral surface and smaller size gives a way to ferric precipitation coves the ore surface hindering the bacterial attachment. The drop in leaching efficiency after few days of inoculation was attributed to the formation of ferric precipitates and the high content of ferric in the study soil. It is claimed that over ferric loading on cells leads to ferric poisoning which leading to the halt of the process However, the observed rate of iron dissolution is found to be very fast within five days of leaching studies. Assay conducted to investigate the influence of sulfate on bioleaching of iron confirms that there is no significant change in the bioleaching rate on sulfate supplement. On Fenton’s oxidation the extracted lateritic iron efficiently played its role as a Fenton’s catalyst in the degradation of herbicides ametryn and dicamba.

## Supporting information

S1 File (DOC)

S1 Graphical abstract (DOC)
