# Supplementary material for: Bioleaching of iron from laterite soil using an isolated Acidithiobacillus ferrooxidans strain and application of leached laterite iron as Fenton’s catalyst in selective herbicide degradation
Source: PLoS One. 2021 Mar 30;16(3):e0243444. doi: 10.1371/journal.pone.0243444 (PMC8009436; doi:10.1371/journal.pone.0243444)
Supplement: S1 File — (DOC) [file pone.0243444.s001.doc]

**S1 File.**

**Highlights**

- Bioleaching of iron from laterite soil using an isolated strain *AcidithiobacillusFerrooxidans* BMSNITK17.
- Investigation of the effect of various parameters like Pulp density, Particle size, pH, Temperature, Shake flask speed on bioleaching of iron from lateritic soil.
- Effect of sulfate addition on bioleaching of iron from lateritic soil.
- Evaluation of catalytic efficiency of leached laterite iron on Fenton’s degradation of selective herbicides Ametryn and Dicamba.
