# Supplementary figures and images for: Bioleaching of iron from laterite soil using an isolated Acidithiobacillus ferrooxidans strain and application of leached laterite iron as Fenton’s catalyst in selective herbicide degradation
Source: PLoS One. 2021 Mar 30;16(3):e0243444. doi: 10.1371/journal.pone.0243444 (PMC8009436; doi:10.1371/journal.pone.0243444)

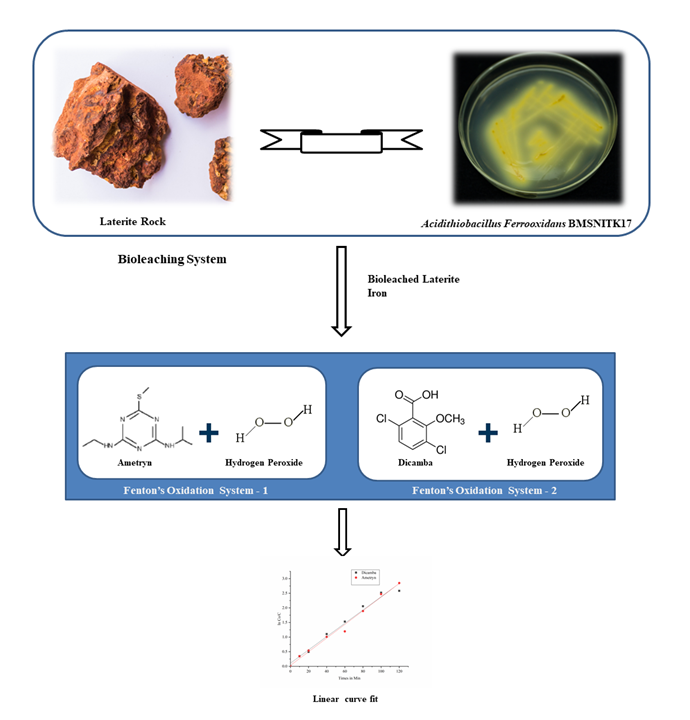

Supplement: S1 Graphical abstract — (DOC) [file pone.0243444.s002.doc]
